# Extensively drug-resistant *Klebsiella pneumoniae* ST307 outbreak, north-eastern Germany, June to October 2019

**DOI:** 10.2807/1560-7917.ES.2019.24.50.1900734

**Published:** 2019-12-12

**Authors:** Sebastian Haller, Rolf Kramer, Karsten Becker, Jürgen A Bohnert, Tim Eckmanns, Jörg B Hans, Jane Hecht, Claus-Dieter Heidecke, Nils-Olaf Hübner, Axel Kramer, Kathleen Klaper, Martina Littmann, Lennart Marlinghaus, Bernd Neumann, Yvonne Pfeifer, Niels Pfennigwerth, Simone Rogge, Katharina Schaufler, Andrea Thürmer, Guido Werner, Sören Gatermann

**Affiliations:** 1 Robert Koch Institute, Department for Infectious Disease Epidemiology, Berlin, Germany; 2 Robert Koch Institute, Division of Nosocomial Pathogens and Antibiotic Resistance, Wernigerode, Germany; 3 Robert Koch Institute, Genome Sequencing Unit, Berlin, Germany; 4 Regional Public Health Authority Mecklenburg-Western Pomerania, Rostock, Germany; 5 University Medicine Greifswald, Greifswald, Germany; 6 National Reference Centre for multidrug-resistant Gram-negative bacteria, Ruhr University Bochum, Bochum, Germany; 7 Institute of Pharmacy, University of Greifswald, Greifswald, Germany

**Keywords:** Enterobacterales, clonal spread, NDM-1, OXA-48, colistin resistance, CTX-M, XDR

## Abstract

From June to October 2019, 17 patients (six infected, 11 colonised) with an extensively drug-resistant (XDR) *Klebsiella pneumoniae* strain were notified from four Western Pomerania medical facilities. The XDR *K. pneumoniae* produced carbapenemases NDM-1 and OXA-48, and was only susceptible to chloramphenicol, tigecycline and cefiderocol. Synergistic activity was observed for the combination of aztreonam plus ceftazidime-avibactam. Genomic analyses showed all isolates belonged to *K. pneumoniae* sequence type 307. Control measures and further investigations are ongoing.

Between June and October 2019, a university hospital, two other hospitals and a rehabilitation clinic in north-eastern Germany (Western Pomerania, Greifswald) were affected by an outbreak of extensively drug-resistant (XDR) *Klebsiella pneumoniae*. A total of 17 patients were infected or colonised. The aim of this short article is to provide detailed information on the epidemiological and microbiological results of this outbreak investigation, and thereby facilitate comparison with similar outbreaks of this emerging Klebsiella strain on international level.

## Outbreak detection and investigation

Following a routine microbiological screening of tracheal secretion on an intensive care unit (ICU) at a university hospital, the first patient with XDR *K. pneumoniae* was detected on 25 June in calendar week 26. The patient was placed in a single room and enhanced barrier precautions were put in place. Contact patients were screened and an admission and exit screening for colonisation with Gram-negative bacteria was performed on the affected ICU in the university hospital. Despite extended precautions, further cases were detected on six wards at the university hospital throughout the following weeks. Under the impression of an ongoing outbreak, intensified measures such as screening, environmental testing and cohorting of cases to a special isolation unit were implemented. In the meantime, patients with XDR *K. pneumoniae* were detected in a primary-hospital, secondary-hospital and rehabilitation clinic in the region.

All isolates from all affected medical facilities were sent to the National Reference Centre (NRC) for multidrug-resistant (MDR) Gram-negative bacteria in Bochum. There the microbiological and molecular analyses of corresponding isolates revealed the production of two carbapenemases, NDM-1 and OXA-48, and colistin resistance.

A case was defined as a person carrying the *K. pneumoniae* outbreak clone as confirmed by molecular typing AND having an epidemiological link to the outbreak.

Cases and outbreak information were reported to the responsible health authorities. Extensive drug resistance, a high proportion of infections as well as challenges in outbreak control led to involvement of the national public health services (in calendar week 38).

**Figure 1:**
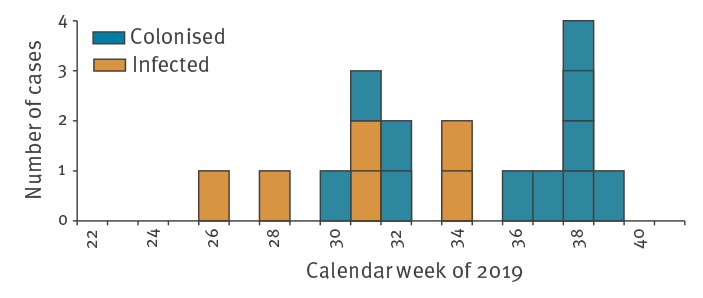
Number of *Klebsiella pneumoniae* ST307 outbreak cases according to date of first detection per calendar week, north-eastern Germany, June–October 2019

**Figure 2:**
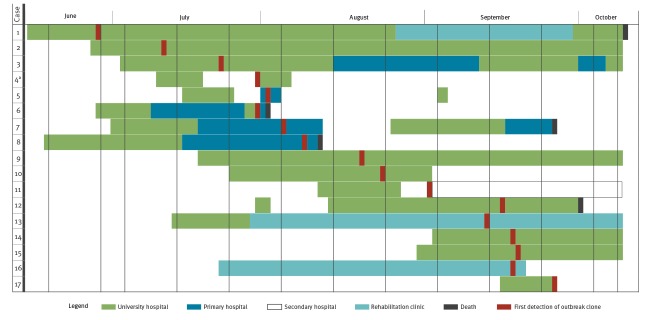
Timeline of *Klebsiella pneumoniae* ST307 outbreak, north-eastern Germany, June–October 2019

Of the 17 cases (Figure 1), two were female and 15 were male, with a median age 70 years (range: 50–84 years). Six cases presented clinical symptoms (sepsis, pneumonia, urinary tract infection), 11 cases were colonised; most had severe underlying diseases. Of the 17 cases, 10 were diagnosed in the university hospital, four in a primary hospital, two in the rehabilitation clinic and one in a secondary hospital (Figure 2). All but one case had been exposed to treatment in the university hospital before the first detection of the outbreak clone. The only case not exposed at the university hospital shared the same room with another case in the rehabilitation clinic (Figure 2). By 15 October 2019, six cases had died apparently from underlying diseases; nonetheless, causal links with the outbreak clone are under further investigation.

Dates of detection are given as known by the Robert Koch Institute.

Time intervals of 10 days between vertical lines. Dates of detection reported as known by the Robert Koch Institute. Case 1 was hospitalised on 7 June 2019 and died on 15 October 2019. A total of six cases died, of which Case 4 died after discharge (^a^date not specified). The first detection of the outbreak strain was on 25 June 2019 (Case 1) and Case 17 was detected on 26 September 2019.

## Microbiological and molecular analyses

Initial antibiotic susceptibility testing (AST) of 10 isolates using semi-automated VITEK 2 (Biomerieux, Nürtingen, Germany) analysis and 96-well plate broth microdilution (Merlin, Bornheim-Hersel, Germany) revealed resistance to a wide range of antibiotics. Testing for common carbapenemase gene families indicated presence of *bla*_OXA-48-like_ and *bla*_NDM_ in the isolates. Furthermore, isolates showed resistance against aztreonam and ceftazidime-avibactam. However, to assess therapeutic options an Etest synergy assay (bioMérieux, Nürtingen, Germany) with ceftazidime-avibactam and aztreonam was used, with results suggesting a synergistic activity. An extended AST showed extensive drug-resistance with susceptibility only to chloramphenicol, tigecycline and cefiderocol, the latter of which is not yet approved for use in European Union/European Economic Area (EU/EEA) countries.

For early discrimination of outbreak and non-outbreak isolates, a loop-mediated isothermal amplification (LAMP) assay (eazyplex SuperBug CRE, AmplexDiagnostics, Gars, Germany) for rapid detection of the carbapenemase genes was included in the university hospital laboratory diagnostic procedure.

The NRC performed molecular typing by *Xba*I-macrorestriction analyses in PFGE. Following the criteria of Tenover et al. [1], analyses revealed that all isolates of the 17 cases belonged to the same outbreak strain (Supplementary Figure S1).

**Figure 3:**
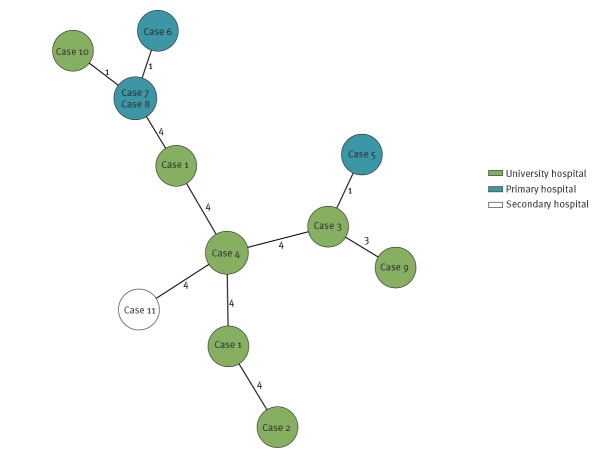
Minimum spanning tree of 13 XDR *Klebsiella pneumoniae* ST307/CT3459 isolates of the outbreak based on allelic profiles, north-eastern Germany, June–October 2019

Thirteen isolates from the first eleven patients were available for whole-genome sequencing (WGS) using the MiSeq System (Illumina, San Diego, United States). Sequence analyses and typing were done using Kleborate tool and other established methods [2-6]. Core genome (cg)MLST analyses with the Ridom SeqSphere^+^  software version 6.0.0 confirmed PFGE results, showing a maximum of 12 allelic differences between isolates (Figure 3). In silico typing revealed that all isolates belonged to sequence type (ST)307 and cgMLST complex type CT3459, with the capsule type K2 (*wzi-173*, *wzc-939*). Sequence analyses apart from carbapenemase encoding genes revealed a broad spectrum of further resistance genes, e.g. *bla*_CTX-M-15,_ *qnrS1* (Table). There was no evidence for the presence of the transmissible colistin resistance gene *mcr-1* or related variants. However, amino acid substitutions were identified in intrinsic two component systems, PmrA/PmrB and PhoP/PhoQ, which have previously been shown to mediate colistin resistance [7].

For each isolate, an allelic profile was determined by cgMLST analysis using the Ridom SeqSphere^+^  software version 6.0.0. These profiles were used to determine both the complex type CT3459 and the genetic distances between isolates. Results visualise relatedness of isolates on core-genome scale as minimum spanning tree (MST). The MST was calculated with the option ‘pairwise ignore missing values’. Each resulting circle represents a distinct allelic profile. Cases from which the isolates were collected are shown for each allelic profile. Two isolates from Case 4 showed identical allelic profiles and were grouped together in a single circle (see also Case 7 and Case 8). Numbers on connecting lines represent allele differences between allelic profiles. Colours of circles indicate for each case the hospital where the outbreak strain was detected for the first time.

**Table:** Resistance genes detected in the *Klebsiella pneumoniae* ST307 outbreak strain using the Kleborate tool, north-eastern Germany, June–October 2019

Affected antibiotic class	Detected genes/gene cluster
**Beta-lactams**	*bla*_NDM-1_
	*bla*_OXA-48_
	*bla*_CTX-M-15_
	*bla*_TEM-1B_
	*bla*_SHV-28-like_
**Aminoglycosides**	*Aac(3)-IIa-like*
	*aph(3”)-Ib*
	*aph(3')-Ia-like*
	*aph(3')-VI*
	*Aph(6)-Id*
	*armA*
**Quinolones**	*oqxA-like*
	*oqxB-like*
	*qnrS1*
	*gyrA 83*
	*parC 80*
**Macrolides**	*mph(A)-like*
	*mph(E)*
	*msr(E)*
**Sulphonamides**	*sul1*
	*sul2*
**Fosfomycin**	*fosA-like*
**Trimethoprim**	*dfrA5*

Comparisons between the 13 isolates and sequence collections from other countries revealed similarity with a Finnish ST307 isolate from a patient with prior hospitalisation in Saint Petersburg, Russia [8]. Although this isolate contained the same resistance genes, there is no evidence of an epidemiological link to the current outbreak. Even though an admission screening for risk groups as patients with prior hospitalisation abroad is routinely performed in Germany, we assume that the primary case was missed. In contrast to the Finnish patient, the index case had no known risk factors. Thus, the reason for the spread into other institutions, in difference to Finland, may have occurred because of unknown colonised cases. A nucleotide sequence from the present outbreak strain has been deposited in the Sequence Read Archive (accession number: PRJNA594546).

## Outbreak control measures

Healthcare facilities, general practitioners and regional medical laboratories of the region were informed about the outbreak by public health authorities and the regional multidrug-resistant organisms (MDRO)-network on 24.09.2019.

In calendar week 38, an extended outbreak meeting with the federal, state and regional public health authorities, the affected facilities and the regional MDRO-network was organised to harmonise case search, follow-up of cases and hygiene measures. From calendar week 39 onwards, all institutions with more than one case performed one-time screening of all patients, prevalence screening, on all wards to make sure that existence of undetected cases was unlikely. In addition, a weekly screening was performed for 6 weeks starting at calendar week 40 on all wards where a case had stayed overnight. A notification of discharged patients that were hospitalised during the outbreak when transmission was not yet controlled, was discussed, but not introduced. Instead, all hospitals in the region were asked to perform an admission screening on patients that were exposed to one of the outbreak facilities from June 2019 onwards. The last transmission was detected in calendar week 39.

An overview about this outbreak with a brief description of the pathogen was published in the weekly German national bulletin on 2 October 2019, and information was internationally shared via the European Centre for Disease Prevention and Control’s (ECDC) Early Warning and Response System (EWRS) and Epidemic Intelligence Information System (EPIS) and the World Health Organization’s (WHO) Global Antimicrobial Resistance Surveillance System-Emerging Antimicrobial Resistance Reporting (GLASS-EAR) [9].

## Discussion

This is the first reported nosocomial outbreak of XDR *K. pneumoniae* ST307 with NDM-1, OXA-48 and CTX-M-15 in Germany. Despite established hygiene precautions, a university hospital, two other hospitals and a rehabilitation clinic in north-eastern Germany were affected. This may suggest that the outbreak clone is highly adapted to the hospital environment. Microbiological analyses of the outbreak strain are ongoing, preliminary results of susceptibility testing to disinfectants does not suggest increased disinfectant resistance.

The outbreak seemed controlled until week 44 as no new transmissions occurred. In calendar week 44, however, Case 18 was detected through admission screening in the university hospital. This case had been hospitalised during July and September on wards of the university hospital with other cases and had not been screened during these stays. Identification of this case indicates that further unknown cases may exist. This highlights the need for close information exchange between referral hospitals and for screening of patients that were exposed to one of the outbreak facilities from June 2019 onwards. A new case was also detected through rectal screening in week 45. This case, Case 19, stayed on the ward where current cases are being cohorted and a recent transmission event seems most likely. Again an extended outbreak meeting with all stakeholders was organised in week 46 and barrier precautions were reinforced. Currently data collection of these two recent cases as well as sequencing of the isolates are being conducted. PFGE analysis of the new isolates shows that these belonged to the outbreak strain. As of calendar week 49, there have been no further cases detected.

The mode of transmission is still under investigation, but person-to-person transmission is most likely the relevant route. In this context, the following two steps are especially valuable: ensuring intensified isolation for cases and intensified case searching through systematic extended screening immediately when an outbreak is suspected, to avoid further unrecognised cases. Other outbreaks with similar pathogens have shown that systematic rectal screening is crucial to identifying colonised patients [10]. Early in-depth analysis of the molecular and phenotypic strain features supports decision making processes for treatment and outbreak control.

The first case (index patient) presented no typical risk factors for *K. pneumoniae* infection such as a recent hospital stay or recent travel, and is therefore unlikely to be the primary case that brought the outbreak strain into the university hospital. As exemplified by the detection of Case 18, there may have been undetected cases, especially during the early phase of the outbreak. All but one case (of the 19 cases) had a history of hospitalisation in the university hospital, which is the only tertiary hospital in the affected region. Transmissions within the other institutions involved cannot be ruled out. Cooperation between institutions and public health authorities should be intensified, e.g. by fostering close, local collaboration between health facilities, health departments, laboratories and further stakeholders in MDRO-networks. This has already been proven to be successful for controlling outbreaks with other XDR pathogens [11].

The emergence of XDR *K. pneumoniae* dramatically limits treatment options. Only a few outbreaks of *K. pneumoniae* with the carbapenemase combination of NDM-1 and OXA-48 have been described [12,13]. Furthermore, presence of both carbapenemases NDM-1 and OXA-48 were identified in other *K. pneumoniae* sequence types [14,15], but rarely in ST307 [16]. However, CTX-M-15-associated *K. pneumoniae* ST307 with OXA-48 and colistin resistance was reported in a Serbian hospital in 2015 [7]. Wyres et al. [17] recently described CTX-M-15-associated *K. pneumoniae* ST307 as a highly successful, globally emerging lineage with remarkable level of plasmid conservation.

Clinical and laboratory staff need to increase vigilance in order to improve early detection of XDR outbreaks. Early extensive screening and a high level of isolation precautions are needed to avoid further spread of these pathogens. As consequence of this outbreak, the detection of an XDR enterobacterial strain in an area with low endemicity emphasizes the need for increased awareness and risk mitigation measures to avoid transmission events.

## Supplementary Data

511000 Supplementary Material
