# Supplementary material for: Extensively drug-resistant Klebsiella pneumoniae ST307 outbreak, north-eastern Germany, June to October 2019
Source: Euro Surveill. 2019 Dec 12;24(50):1900734. doi: 10.2807/1560-7917.ES.2019.24.50.1900734 (PMC6918589; doi:10.2807/1560-7917.ES.2019.24.50.1900734)

Supplementary Material

This supplementary material is hosted by *Eurosurveillance* as supporting information alongside the article “Extensively drug-resistant Klebsiella pneumoniae ST307 outbreak, in north-eastern Germany, 2019”, on behalf of the authors, who remain responsible for the accuracy and appropriateness of the content. The same standards for ethics, copyright, attributions and permissions as for the article apply. Supplements are not edited by *Eurosurveillance* and the journal is not responsible for the maintenance of any links or email addresses provided therein.

Supplementary Figure S1: Pulsed-field gel electrophoresis (PFGE) profiles from 17 isolates belonging to the Klebsiella pneumoniae ST307 outbreak in north-eastern Germany 2019

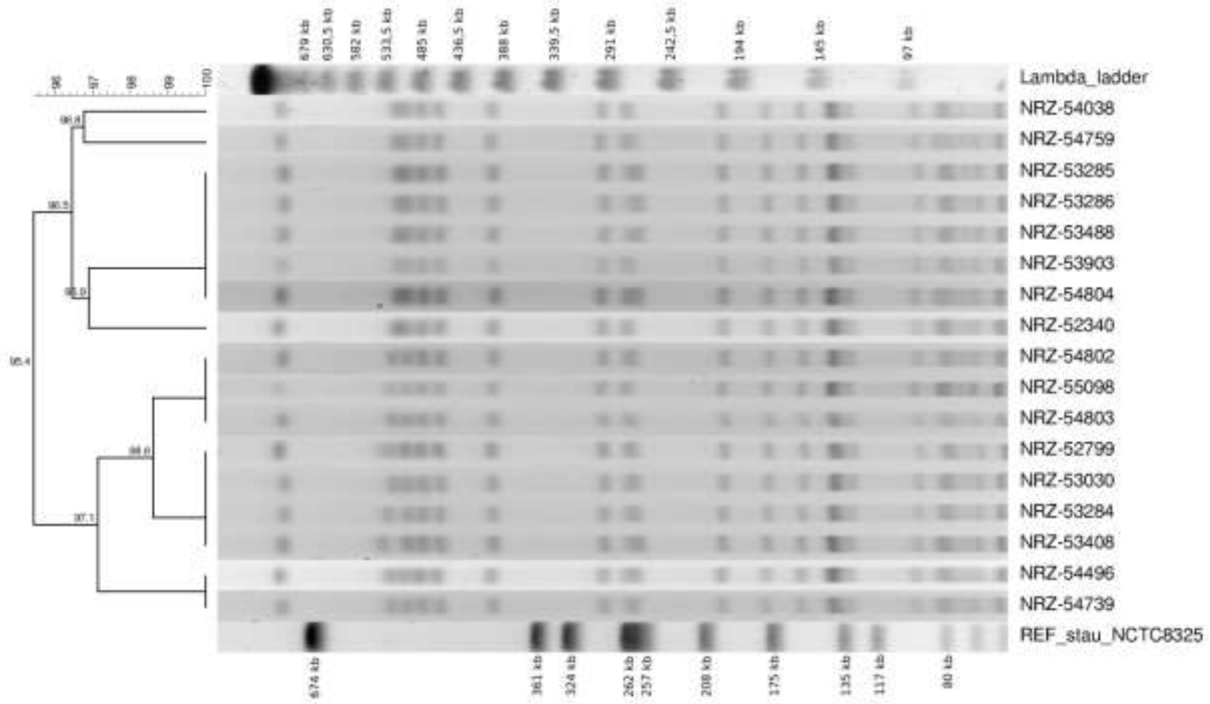

Supplement: Supplementary Material [file 19-00734_SupplementaryMaterial.pdf]
